# Multifaceted Nutrition Intervention for Frail Elderly in the Community: Protocol of a Randomized Controlled Trial (The MINUTE Study)

**DOI:** 10.3390/nu17203213

**Published:** 2025-10-13

**Authors:** Yaxin Han, Haohao Zhang, Meng Sun, Yuxin Ma, Yahui Tu, Jiajing Tian, Rui Fan, Wenli Zhu, Zhaofeng Zhang

**Affiliations:** 1 Department of Nutrition and Food Hygiene, School of Public Health, Peking University, Haidian District, Beijing 100191, China; hanyaxin@bjmu.edu.cn (Y.H.);; 2 Beijing’s Key Laboratory of Food Safety Toxicology Research and Evaluation, Beijing 100191, China; 3 Institute of Medical Technology, Peking University Health Science Center, Beijing 100019, China

**Keywords:** frailty, randomized controlled trial, multifaceted nutrition intervention, healthy aging, community, frail elderly

## Abstract

Background: The rapid aging of China’s population poses significant challenges, particularly in public health and medical services. Frailty, a reversible geriatric syndrome, is a critical intervention target for disability prevention among older adults. Objective: We hypothesize that both intervention groups will demonstrate significant improvements in Short Physical Performance Battery (SPPB) scores compared to the control group, and that these improvements will be accompanied by parallel reductions in inflammatory markers and beneficial alterations in the gut microbiota. Methods: The MultIfaceted NUtrition inTervention for frail Elderly (MINUTE) trial is a randomized, parallel-group controlled trial. In Beijing, China, 315 frail older adults were recruited and randomly assigned to 3 groups: a control group receiving routine community health management only, multifaceted nutrition intervention group, and a multifaceted nutrition and exercise combined intervention group, each comprising 105 participants. The study consists of a three-month intervention period followed by a nine-month follow-up. During the three-month intervention period, the control group receives routine community health management, while the multifaceted nutrition intervention group receives daily dietary guidance, personalized nutrition consultations, and health education. Additionally, the combined intervention group receives exercise interventions in addition to the nutritional components. After the three-month intervention, all three groups will be followed up for nine months to assess the sustainability of the study. Results: The primary outcomes are the changes in the SPPB scores. The secondary outcomes include frailty scores, intrinsic capacity, malnutrition risk, frailty recovery rates, serum differential metabolites, inflammatory factors, and gut microbiota changes. This study aims to establish a scalable and sustainable pathway for frailty prevention among community-dwelling older adults in China and provide valuable insights to inform strategies for healthy aging. Trial registration: This study is conducted in accordance with the Declaration of Helsinki and approved by the Peking University Institutional Review Board (IRB00001052-23178) on February 3, 2024, with all amendments subject to prior review and approval. Informed consent is obtained from participants, and findings will be disseminated through peer-reviewed publications, conference presentations, and summaries for school staff and participants. ClinicalTrials.gov (NCT06547593) registered 30 July 2024.

## 1. Introduction

The global elderly population is undergoing rapid expansion, with the age group of 65 and older experiencing the most pronounced growth. China’s population is aging rapidly, and this demographic shift is becoming increasingly prevalent across the nation [1,2]. Research findings suggest that the risks of falls, disability, delirium, hospitalization, and premature mortality among the elderly are progressively rising in tandem with the aging process [3]. This trend exacerbates the burden on caregivers and healthcare systems, thereby intensifying the challenges of an aging society [4,5]. By 2050, projections indicate that individuals aged 65 and older will comprise one-third of the total population [6]. The swift demographic shift toward an aging population in China [7], characterized by a substantial base of elderly individuals, is rapid and outpacing the country’s current level of socioeconomic development [8]. This demographic transition poses significant challenges to multiple sectors of Chinese society [9], particularly to public health and medical services. Consequently, addressing the challenges associated with an aging population has been prioritized as a national policy in China.

The overall health status of China’s elderly population is a cause for concern. Approximately 44 million older adults experience partial or complete disability, which places a substantial strain on their families [10]. This not only casts a long shadow over their own lives [11] but also places a significant burden on their families and society [12,13]. Disability has become a pressing public health issue that demands the attention of the entire nation [14].

Frailty, a geriatric syndrome marked by heightened vulnerability to stressors, reflects a state of reduced physiological reserve and impaired function across multiple organ systems, resulting in increased dependency and mortality [15,16]. A study based on data from 62 countries indicates that the prevalence of frailty is 24%, while the prevalence of pre-frailty can be as high as 49% [17]. Frailty serves as a critical intervention target for disability, as it is reversible. The screening and intervention of frailty are essential for preventing disability among older adults and promoting their vitality. Addressing frailty is crucial for enhancing the quality of life and independence of the elderly, thereby alleviating the societal burden associated with an aging population [18,19].

Frailty is increasingly recognized in geriatric medicine, with more and more guidelines and expert consensus being developed. A growing number of frailty intervention studies are also being conducted. Existing frailty intervention studies encompass physical activity [20,21], nutritional supplementation [22], cognitive training [23,24], comprehensive geriatric assessments [25], physical activity combined with nutritional supplementation [22], and multicomponent interventions (e.g., physical activity, nutritional supplementation, educational activities, group dining, personalized nutrition advice, and social support) [26]. A growing body of evidence has demonstrated the potential of these interventions in managing frailty. Physical exercise, particularly resistance and multicomponent training, has consistently been shown to improve muscle strength, gait speed, and physical performance, which are core components of the frailty phenotype [20,21]. However, the long-term sustainability of these benefits often requires ongoing exercise commitment, and adherence can be challenging [27]. Nutritional supplementation, primarily focusing on protein and vitamin D, has demonstrated efficacy in addressing sarcopenia—a key element of frailty—and reducing malnutrition risk [28]. Nevertheless, the effects of isolated nutritional supplementation on broader frailty metrics are often modest, suggesting that simply addressing nutrient deficits may be insufficient [29]. Multicomponent interventions, which integrate physical activity, nutritional support, and other elements like cognitive training, represent a more holistic approach. Landmark trials, such as the SPRINTT project, have shown that such integrated interventions can significantly reduce the risk of mobility disability in frail older adults [22]. Similarly, the VIVIFRAIL study demonstrated that a tailored multicomponent exercise program improved functional capacity in frail, pre-frail, and robust older adults [30]. Despite their promise, these comprehensive approaches often face challenges in real-world implementation, including complexity, high resource demands, and limited evidence of scalability in diverse community settings, particularly within the specific socio-cultural context of China [31].

However, current research has several limitations when considered for application in China. Firstly, most studies remain limited to the guideline level, significantly lagging in practical application, failing to address the individualized needs of the elderly, overlooking the considerations of multiple stakeholders, and lacking clear, actionable, and feasible practical pathways. Secondly, existing guidelines predominantly focus on foreign contexts and do not align with Chinese characteristics, lacking research evidence specifically tailored to the Chinese population. There is also a dearth of research on the prevention and management of frailty among the elderly in community settings. Therefore, there is a pressing need to conduct a multidimensional nutritional intervention study on frailty among older adults in China. Against this backdrop, we propose the initiation of the MultIfaceted NUtrition inTervention for frail Elderly (MINUTE) project, which aims to bridge the aforementioned gaps and provide a valuable pathway for healthy aging.

## 2. Methods

### 2.1. Aims

The primary aim of the MINUTE trial is to evaluate the effects of a multifaceted nutrition intervention and a combined nutrition and exercise intervention on physical performance (SPPB score) among frail older adults.

Primary hypotheses: We hypothesize that (1) after the 3-month intervention, both the multifaceted nutrition intervention group and the combined intervention group will show a greater improvement in SPPB score compared to the control group. (2) The improvements in physical performance and frailty in the intervention groups will be mediated by concurrent reductions in systemic inflammation and beneficial alterations in the gut microbiota composition.

Secondary hypotheses: We further hypothesize that the multifaceted nutrition and exercise combined intervention will be superior to the nutrition-only intervention in improving physical performance.

### 2.2. Study Design

**Figure 1:**
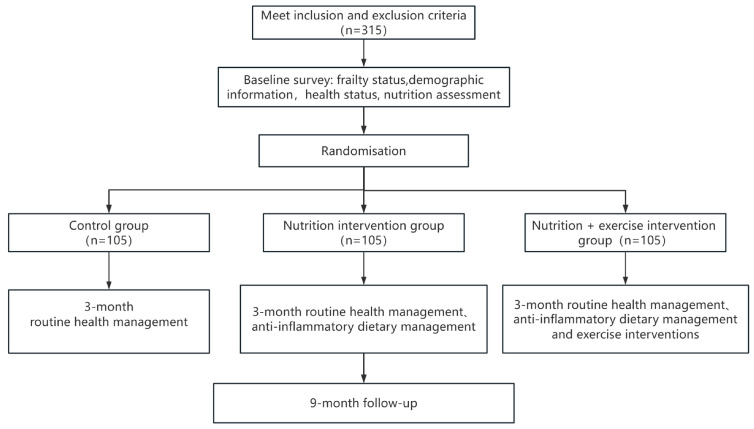
Study flowchart inclusion to follow-up.

**Figure 2:**
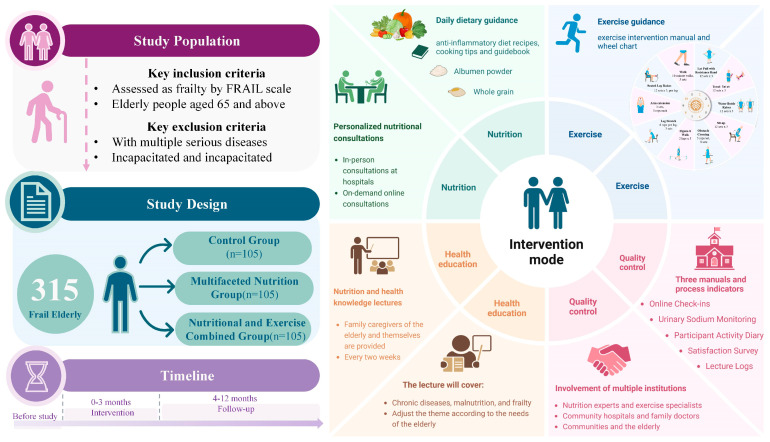
Detailed overview of the study.

The MINUTE trial is a randomized, parallel-group, controlled study involving a total of 315 participants. Participants will be randomly assigned to one of three groups: a control group that will receive routine community health management and serve as the reference for comparing the effectiveness of the active interventions, a multifaceted nutrition intervention group, or a multifaceted nutrition and exercise combined intervention group. Each group will include 105 participants and will receive a different intervention over a 3-month period, followed by a 9-month follow-up. Upon study completion, between-group comparisons will be conducted to evaluate the differential effects of the interventions. Figure 1 illustrates the study flowchart from participant inclusion through the follow-up phase. Figure 2 (graphical abstract) provides a detailed overview of the study population, design, and interventions. The trial has been registered on ClinicalTrials.gov (Identifier: NCT06547593).

### 2.3. Recruitment of Participants

Participants will be recruited from the senior community. We will collaborate with the Community Health Service Center to disseminate recruitment information through community physicians and neighborhood committees. Recruitment will be conducted in several stages to ensure a thorough and systematic approach:

(1) Initial contact and information dissemination: Community physicians and neighborhood committees will identify potential participants who meet the basic age criteria (aged 65 years or older). They will provide these individuals with detailed information about the study, including its purpose, procedures, risks, and benefits. This information will be delivered through flyers, community announcements, and face-to-face consultations.
(2) Screening and pre-selection: Potential participants will be invited to attend a preliminary screening session at the Community Health Service Center. During this session, community physicians will conduct a brief health assessment and administer the FRAIL scale to determine the frailty status. Individuals who are assessed as frail by the FRAIL scale will be considered for further evaluation.
(3) Informed consent and detailed assessment: Eligible individuals will be provided with a detailed explanation of the study procedures and will be asked to provide written informed consent. Following consent, a comprehensive baseline assessment will be conducted by trained healthcare professionals. This assessment includes a review of medical history, physical examination, and evaluation of functional and mental health status.

Inclusion criteria include the following:

(1) Assessed as frailty by the FRAIL scale. The FRAIL scale is a simple 5-item screening tool where frailty is defined as meeting 3 or more of the following criteria: fatigue, resistance (inability to climb one flight of stairs), ambulation (inability to walk 100 m), illnesses (≥5 comorbidities), and loss of weight (>5% in the past year).
(2) Aged 65 years or older.
(3) Able to provide written informed consent, after receiving a detailed explanation of the study purpose, procedures, risks, and benefits.

Exclusion criteria include the following:

(1) Major comorbidities: History of major comorbidities (e.g., stroke, myocardial infarction, active cancer, or severe cardiocerebrovascular disease) that may confound study results or pose risks to participants.
(2) Significant functional impairments: Participants with disability, dementia, severe visual or hearing impairments, or physical activity impairment will be excluded. These impairments will be assessed through standardized functional tests (e.g., Mini-Mental State Examination for cognitive function, visual acuity tests, and hearing assessments) and physical performance tests (e.g., gait speed and balance tests). The exclusion will be based on the potential for these impairments to limit participants’ ability to complete the study protocol or accurately report outcomes.
(3) Moderate to severe mental health conditions: Participants with moderate to severe anxiety or depression will be excluded. Mental health status will be evaluated using validated screening tools (e.g., the Geriatric Depression Scale and the State-Trait Anxiety Inventory). The exclusion will be based on the potential for these conditions to affect participants’ ability to comply with the study protocol or accurately report outcomes.

### 2.4. Randomization, Allocation Concealment, and Blinding

After providing consent and completing the baseline assessment, participants will formally enter the study and be randomly assigned to the control group, the multifaceted nutritional intervention group, or the multifaceted nutrition and exercise combined intervention. The randomization sequence is generated by a statistician from Peking University using computer-generated random numbers. Randomization will be performed in a 1:1:1 ratio using computer-generated random numbers. There will be no blocking or stratification. The allocation will be concealed through central randomization performed by an investigator who is not involved in the assessment or recruitment, and the recruitment personnel will not have access to the group assignment tables. Given the nature of the behavioral interventions, it is not possible to blind the participants or the staff delivering the interventions (nutritionists and exercise specialists). Therefore, this is an assessor-blinded trial. Outcome assessors (staff responsible for administering the physical and cognitive tests) and data analysts will be blinded to group assignment throughout the trial. Participants will be instructed not to reveal their intervention allocation to the outcome assessors. The blinding status of the assessors will be evaluated at the end of the study by asking them to guess the group assignment of the participants.

## 3. Intervention

Table 1 outlines the intervention content for each group.

### 3.1. Control Group

The control group will receive routine community health management only, which represents the standard level of care currently available for frail older adults in the community. This includes monthly health education lectures on general topics (e.g., chronic disease prevention), provision of standard health education materials, and access to basic health counseling services. Critically, the control group will not receive the study’s active intervention components, such as the personalized nutritional consultations, anti-inflammatory diet guidance, provision of protein powder and whole grains, structured exercise guidance, or the intensive bi-weekly health education lectures specific to frailty and malnutrition that are delivered to the intervention groups.

### 3.2. Multifaceted Nutrition Intervention

In this group, a three-month multifaceted nutrition intervention will be conducted. All nutritional interventions and recommendations will be provided to participants based on professional discussions and reviews by nutrition experts from the School of Public Health at Peking University. The specific content is as follows:

(1) Daily dietary guidance: Participants will receive an anti-inflammatory dietary menu (Supplementary Table S1) designed based on the Chinese Dietary Guidelines for the Elderly (2022 Edition) and principles from the Dietary Inflammatory Index [32,33]. The diet emphasizes the intake of fruits, vegetables, whole grains, nuts, and fatty fish while reducing refined carbohydrates and saturated fats. Additionally, we will provide participants with a daily nutritional supplement containing 10 g of whey protein to address the high prevalence of sarcopenia in frail elders and support muscle protein synthesis [34], along with 50 g of whole grains to increase dietary fiber and phytochemical intake, which are associated with improved gut microbiota and reduced systemic inflammation [35,36].

(2) Personalized nutritional intervention: In response to specific health issues and diseases, we have established fixed consultation times at community hospitals, allowing elderly individuals to seek advice at designated intervals if they have health-related needs. Furthermore, we will offer on-demand online consultations with registered dietitians to promptly address the nutrition and health concerns of the elderly.

(3) Health education: Every two weeks, nutrition and health knowledge lectures (Table 2) will be conducted for elderly family caregivers and the elderly themselves, covering topics such as chronic diseases, malnutrition, and frailty.

### 3.3. Multifaceted Nutrition and Exercise Combined Intervention

**Figure 3:**
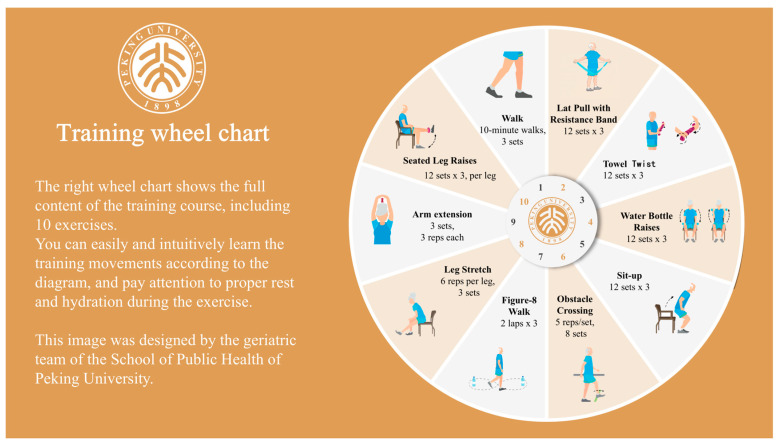
Training wheel chart.

In addition to the aforementioned nutritional measures, exercise interventions will also be included, guided by experts from the Physical Education Department at Peking University. We will provide an exercise intervention manual and a wheel diagram, guiding the elderly to follow a weekly schedule with three days dedicated to resistance exercise sessions and four days for walking, combining resistance and aerobic exercises in a scientifically alternated manner. The weekly resistance exercise program, conducted three times a week, includes eight exercise sessions: twisting towels, lifting water bottles, sit-to-stand training, obstacle crossing, walking in an 8-shape pattern, leg extensions, arm extensions, and seated straight leg raises. These sessions provide comprehensive physical training guidance for the elderly, with details available in Figure 3 and the exercise intervention manual.

To ensure the correctness and safety of the exercises, and to enhance adherence, we will implement a multifaceted support system rather than relying solely on unsupervised self-practice. This includes the following:

(1) Initial supervised instruction: Prior to starting the home-based program, each participant will attend a one-on-one or small-group session with a trained exercise specialist. During this session, the specialist will demonstrate each exercise, ensure the participant can perform it with correct form, and provide personalized feedback.
(2) Structured guidance materials: Participants will receive a detailed, illustrated exercise manual and a training wheel chart (Figure 3) that clearly outlines the weekly schedule, sets, and repetitions, making the program easy to follow at home.
(3) Ongoing remote support: Participants will have weekly access to an exercise specialist via an online platform or telephone to ask questions, report difficulties, and receive motivational support.
(4) Adherence monitoring: Participants will be asked to maintain a simple exercise diary to log their completed sessions. This will be reviewed weekly by the research team to monitor adherence and provide timely reminders if needed.
(5) Integration with community healthcare: To leverage the existing community healthcare network, community hospitals and family physicians will be engaged to provide ongoing supervision. During their routine weekly interactions or health consultations, they will specifically inquire about the participants’ exercise progress, record their adherence, and offer encouragement. This integrates the intervention into the participants’ regular healthcare, providing an additional layer of personal contact and accountability.

### 3.4. Quality Control of the Intervention

(1) To ensure the fidelity and successful implementation of the intervention while prioritizing the safety of the elderly, we will mobilize the involvement of multiple institutions. Nutrition experts offer personalized consultation and guidance on dietary needs, while exercise specialists provide tailored recommendations at varying intensity levels. Community hospitals contribute by offering examination venues and equipment, and family doctors are tasked with ensuring the safety of the elderly. Additionally, staff from senior living communities or care centers assist in the project’s execution, and the elderly actively engage in feedback and communication, sharing their thoughts and experiences.
(2) Three manuals (“Nutrition Intervention Manual for Older Adults”, “the Staff Manual for the Nutrition Intervention Program for Older Adults”, and “Exercise Intervention Manual”) will be used to implement and manage this complex intervention. The manuals detail the responsibilities of study personnel at each stage of the project. The manuals also describe in detail the work processes for implementing each component of the intervention, i.e., who, when, how, and to what extent specific intervention elements are to be delivered.
(3) Throughout the implementation process, project executors will conduct regular interviews and exchanges with the elderly, promptly conveying their thoughts and feedback to the project leaders. Periodic meetings will be held to consolidate the opinions of all stakeholders involved in the project, allowing for the continuous optimization and improvement of any emerging issues.
(4) We will employ a comprehensive set of measures to collect process indicators and ensure adherence to the intervention, as follows:

- Participant activity diary: Records of frail individuals completing nutritional or exercise sessions as prescribed are maintained.
- Satisfaction survey: Frail older adults will be asked to rate their satisfaction with the weekly interventions using smiley faces.
- Online check-ins: Daily check-in frequency and time periods are recorded by designated personnel, detailing the specific daily meals and exercise routines.
- Lecture logs: Records of attendance at each online and offline lecture are maintained, forming a participant log.
- Urinary sodium monitoring: Urine samples from frail elderly individuals will be collected before and after the intervention to monitor urinary sodium levels, assessing dietary adherence.

## 4. Outcomes

Table 3 describes the study outcomes, including when and how the study outcomes will be evaluated. Baseline measurements will be conducted for all three groups. Follow-up measurements will be conducted at 3 months (end of intervention), 6 months, and 12 months (end of follow-up) after the baseline measurement. The primary and secondary outcomes will be assessed by comparing changes from baseline within and between groups.

### 4.1. Primary Outcome

The primary outcome is physical performance, assessed by the change in the Short Physical Performance Battery total score from baseline to the three-month assessment (time point). The SPPB is a well-validated tool ranging from 0 (worst) to 12 (best), assessing balance, gait speed, and lower limb strength. The change will be analyzed as a continuous variable, and the between-group difference will be expressed as the mean difference with a 95% confidence interval.

### 4.2. Secondary Outcomes

(1) Frailty status, assessed by the change in the Frailty Index score from baseline to the 3-month, 6-month, and 12-month assessments. The change will be analyzed as a continuous variable. Changes in frailty trajectories, as measured by the Frailty Index.

(2) Changes in intrinsic capacity: the intrinsic capacity is measured using the World Health Organization’s (WHO) Integrated Care for Older People (ICOPE) approach.

(3) Malnutrition risk assessment, measured by the Malnutrition Risk Assessment Form for older adults.

(4) Frailty recovery rates, defined as the proportion of participants transitioning from a frail to a pre-frail or robust state.

(5) Cognition function is evaluated using the Mini-Mental State Examination (MMSE).

(6) Sleep condition is evaluated using the Pittsburgh Sleep Quality Index.

(7) Participant satisfaction and acceptability are evaluated using a self-administered scale.

(8) Serum protein: serum total protein, albumin, and transferrin.

(9) Urine 8-hydroxyguanosine (8-oxo-Gsn): urine oxidation marker 8-hydroxyguanosine (8-oxo-Gsn) level.

(10) Serum inflammatory factors: C-reactive protein (CRP), interleukin-6 (IL-6), tumor necrosis factor-α (TNF-α), insulin-like growth factor-1 (IGF-1), C-X-C-motif chemokine 10 (CXCL10), and chemokine C-X3-C-based ligand 1 (CX3CL1) in serum using ELISA.

(11) Changes in serum differential metabolites: using nontargeted metabolomics methods to examine the changes in serum differential metabolites in each group before and after intervention and explore the mechanism of intervention.

(12) Changes in gut microbiota: using the 16S sequencing method to detect changes in gut microbiota and differential bacterial genera among groups.

### 4.3. Biological Sample Collection and Handling

Biological samples will be collected at baseline and upon completion of the three-month intervention following an overnight fast. Approximately 10 mL of venous blood will be drawn into serum separator tubes and allowed to clot at room temperature for 30 min, followed by centrifugation at 3000 rpm for 10 min to isolate serum. Concurrently, first-morning void urine samples (approximately 10 mL) will be collected by participants using sterile containers. The resulting serum and urine supernatant will then be aliquoted into 1.5 mL cryovials under standardized protocols. All aliquots will be immediately frozen and stored at −80 °C in the laboratory of Peking University School of Public Health until subsequent batch analysis.

Serum samples will be analyzed for inflammatory biomarkers—including CRP, IL-6, TNF-α, IGF-1, CXCL10, and CX3CL1—using commercially available ELISA kits. Non-targeted metabolomic profiling of serum will be conducted via liquid chromatography–mass spectrometry (LC-MS), while urinary 8-oxo-Gsn will be quantified using ultra-performance liquid chromatography–tandem mass spectrometry (UPLC-MS/MS). Gut microbiota composition will be assessed through 16S rRNA gene sequencing performed on fecal samples collected at home by participants using provided kits and returned to the laboratory under cold chain conditions.

Any residual biological samples will be destroyed after the completion of all planned analyses. The informed consent process includes explicit provision for obtaining participant permission regarding the future use of de-identified samples for additional research related to aging and nutrition.

### 4.4. Adverse Events

An adverse event (AE) is defined as any untoward medical occurrence in a participant during the study period, regardless of its causal relationship with the intervention. All AEs will be monitored throughout the study and systematically collected during the monthly health counseling, bi-weekly lectures, and at each assessment time point (3, 6, and 12 months) using open-ended questions and specific inquiries about common issues, such as falls, musculoskeletal pain, gastrointestinal discomfort, and allergies.

AEs will be graded for severity (mild, moderate, and severe) and their relationship with the intervention (unrelated, possibly related, and probably related) by the principal investigator. All AEs will be recorded in a standardized form. Serious adverse events (SAEs) will be reported to the PKU IRB within 24 h of awareness.

### 4.5. Sample Size Estimation

The sample size calculation was based on the anticipated effect of the intervention on the change in the Short Physical Performance Battery (SPPB) score, which is the primary outcome of this trial.

Based on the results of previous randomized controlled trials [22,37], a multifaceted nutrition and exercise intervention is expected to yield a mean between-group difference of approximately 1.4 points in SPPB score change after 3 months, compared to the control group. The pooled standard deviation (SD) of the SPPB score change is estimated to be 2.5 points.

At α = 0.05, β = 0.1 and k = 3 were used. For a three-group superiority trial, the required sample size per group was calculated:

$$n=\frac{(k-1)(Z_{1-\alpha /2}\;+\;Z_{1-\beta }{)}^{2}\times s^{2}}{\sum_{i=1}^{k}(\mu_{i}-\mu {)}^{2}}$$

Accounting for an anticipated 10% loss to follow-up, the sample size was increased to 105 participants per group, resulting in a total sample size of 315 participants.

### 4.6. Statistical Analyses

This study will employ the Intention-to-Treat (ITT) and Per-Protocol (PP) analysis sets to ensure that all randomized study subjects remain in their assigned groups. The Intention-to-Treat population will include all randomized participants, analyzed according to their assigned group. The Per-Protocol population will include only those participants who completed the intervention as prescribed and had no major protocol deviations. For missing quantitative data, the last observation carried forward method will be used. When the results of the ITT and PP analyses are consistent, the ITT results will be presented. When the results differ, the differences will be discussed. The variables will be analyzed using SPSS V.23.0 software. All statistical tests will be two-sided at the 5% level of significance. Continuous variables with a normal distribution will be shown as the mean and SD and those with an abnormal distribution will be presented as the median and IQR. Categorical variables will be expressed as a percentage or constituent ratio. Baseline characteristics will be presented for each group using descriptive statistics (means and standard deviations for normally distributed continuous data, medians and interquartile ranges for non-normally distributed data, and frequencies and percentages for categorical data). Mixed-model repeated-measures analysis of variance with adjustment for baseline characteristics will be performed for different measurement time points between groups, and interaction between groups and measurement time. For continuous outcomes, we will report the pre- and post-intervention means for the intervention and control groups, as well as the model-adjusted mean differences between groups. For binary outcomes, we will report the pre- and post-intervention percentages for the intervention and control groups, along with the adjusted odds ratios (ORs) between groups. The 95% confidence intervals (CIs) and associated *p*-values will be calculated. We will use nontargeted metabolomics methods to examine the changes in serum differential metabolites in each group before and after the intervention and explore the mechanism of intervention. We will use 16S rRNA gene sequencing to detect changes in gut microbiota and differential bacterial genera among groups. To address the second primary hypothesis regarding the potential mechanisms, correlation analyses (Spearman’s or Pearson’s correlation) and mediation analysis will be performed to examine the relationships between changes in clinical outcomes (SPPB score and frailty) and changes in biomarker levels (inflammatory markers and microbial abundance). Subgroup analyses will be conducted to investigate whether the effects of the intervention vary across different demographic and clinical subgroups.

## 5. Discussion

The MINUTE trial is designed to investigate the effects of multidimensional nutritional interventions on the physical performance and frailty trajectory changes in older adults with frailty, contributing to enhanced physical function and the development of tailored intervention approaches for the Chinese population. These interventions are expected to provide practical strategies for the prevention and management of frailty among older adults in China, offering effective community intervention pathways for healthy aging.

Furthermore, this study will employ metabolomics, gut microbiota analysis, and inflammatory biomarker measurement to elucidate the underlying mechanisms of frailty and the intervention’s effects. Additionally, we will monitor multiple outcome indicators, encompassing the intrinsic capacity, risk of malnutrition, and recovery rate of frailty, offering a comprehensive perspective on the health status of the elderly. This multidimensional approach establishes actionable pathways for nutritional interventions against frailty, effectively bridging the gap between theoretical guidelines and practical implementation. Moreover, this project actively engages with multiple stakeholders, offering a scalable, sustainable, and replicable comprehensive intervention strategy for healthy aging in China.

This study offers several advantages. Firstly, it features a large sample size and long-term follow-up, employing a randomized controlled trial design. Through nearly a year of follow-up, we will assess the effectiveness and sustainability of the intervention pathway. Secondly, in China, nearly 96% of the elderly reside at home or in community settings. Our community-based project offers tailored strategies specifically designed for these home-based older adults. Thirdly, this research has strong operability and popularization. This project engages multiple stakeholders, holding meetings and discussions with various interest groups, integrating diverse perspectives, aligning with the interests of organizations and considering the needs of all parties. Nutrition experts provide nutritional guidance, exercise specialists offer exercise interventions, community hospitals conduct health examinations, family doctors ensure participant safety, senior living communities are responsible for promotion and publicity, and the elderly themselves contribute their suggestions and feedback. Fourthly, to ensure the reliability of the study, we will implement a comprehensive set of quality control measures, encompassing participant activity diaries, satisfaction surveys, online check-ins, lecture logs, and additional measures.

However, this study also has several potential limitations. Firstly, this study employs a single-blind design, as the nature of the interventions precludes blinding the staff administering the interventions or the participants themselves. Participants will be instructed not to disclose their intervention status to the assessors. However, this does not affect the progress or credibility of the project, as it reflects the participants’ true circumstances. Secondly, our conclusions regarding rural areas are conservative and cautious. Our model may not be directly applicable to rural settings, and the generalizability of our results should be approached with care. In the future, we plan to further explore this research in rural areas to gain more insights.

## Figures and Tables

**Table 1:** Description of the intervention content for each group.

Group	Intervention Components	Personnel	Frequency
Control Group	1. Routine community health lectures - Health education lectures - Provision of health education materials - Health counseling services	Trained project staff	Monthly
Multifaceted Nutrition Intervention	1. Daily dietary guidance - Provision of anti-inflammatory diet recipes, cooking tips, and a guidebook - Daily provision of 10 g of whey protein powder and 50 g of whole grains	Trained nutrition specialists	Weekly
	2. Personalized nutritional consultations - Integration of fixed-time, in-person hospital consultations with on-demand online consultations	Intelligent app/programs	At any time
	3. Health education - Lectures on chronic diseases, malnutrition, and frailty	Trained nutrition specialists	Every two weeks
Multifaceted Nutrition and Exercise Combined Intervention	Includes all components from the Multifaceted Nutrition Intervention group, PLUS: 4. Exercise guidance - Provision of an exercise intervention manual and wheel chart - Distribution of elastic resistance bands for resistance exercises	Trained sports specialists	Weekly

**Table 2:** Outline of the health education activities for the elderly.

**No.**	**Activities**	**Content (Lecture Theme)**
The first month		
1	Lecture 1	Nutritional Status and Common Health Issues in the Elderly
2	Lecture 2	Unlocking the Health Code: Understanding Nutrients
Qualitative interviews: satisfaction survey and feedback		
The second month		
3	Lecture 3	Unlocking the Health Code: Recognizing the Nutritional Value of Foods
4	Lecture 4	Focus on Nutritional Screening: How the Elderly Can Identify Malnutrition
Qualitative interviews: satisfaction survey and feedback		
The third month		
5	Lecture 5	How the Elderly Should Plan Their Three Meals a Day
6	Lecture 6	To Live Longer, Overcome ‘Physical Frailty’—A New Perspective on Elderly Health Management
Qualitative interviews: satisfaction survey and feedback		
The fourth month		
7	Lecture 7	A Healthy Life, ‘Muscle’ is Essential—Nutritional Management of Sarcopenia
8	Lecture 8	Scientific Diet: Helping You ‘Keep Your Memory’
Qualitative interviews: satisfaction survey and feedback		

**Table 3:** Schedule of assessments and outcome measures.

Measurements	Time Points				Instrument/Method of Assessment	Outcome Variables
	Baseline	3 Months	6 Months	12 Months		
Physical examination						
Height	Yes	Yes	Yes	Yes	Stadiometer	BMI
Weight	Yes	Yes	Yes	Yes	Lever scale	
Waist/Calf circumference	Yes	Yes	Yes	Yes	Tape	Waist/Calf circumference
Sebum	Yes	Yes	Yes	Yes	Sebaceous pliers	Body fat percentage
Body composition analysis	Yes	Yes	Yes	Yes	Body composition analyzer/bioelectrical impedance analysis technique	Body fat mass, body fat percentage, defatted body weight, muscle mass, body water, protein, inorganic salts, and other data
(Strength of one’s) Grip	Yes	Yes	Yes	Yes	Grip strength meter/adjust for palm size, measure with index finger second joint at a right angle, maximum effort	Grip strength measurements
Simple Physical Performance Based Assessment (SPPB)						
Balance testing	Yes	Yes	Yes	Yes	Timekeeping device/includes balancing in 3 different difficulty positions: feet together, half tandem standing, and tandem standing.	Test scores
3 or 4 meter pace test	Yes	Yes	Yes	Yes	Timekeeping device/walk at maximum speed. Record the shortest of two trials.	Test scores
Timed Sit-Stand Test	Yes	Yes	Yes	Yes	Timekeeping device/assess lower limb strength in elders by timing 5 rapid sit-to-stand cycles with a stopwatch.	Test scores
Frailty status						
Fried Frailty Phenotype	Yes	Yes	Yes	Yes	Scale questionnaire	Scale score
Tilburg Frailty Indicator	Yes	Yes	Yes	Yes	Scale questionnaire	Scale score
Frailty trajectories	Yes	Yes	Yes	Yes	Changes in frailty trajectories before and after the intervention, as measured by the Frailty Index	
Frailty recovery rates	Yes	Yes	Yes	Yes	Difference in frailty scores before and after intervention	
Nutritional status						
Malnutrition risk assessment	Yes	Yes	Yes	Yes	Scale questionnaire	Scale score
Nutritional literacy assessment for the elderly	Yes	Yes	Yes	Yes	Scale questionnaire	Scale score
Metabolomics						
Serum protein	Yes	Yes		Yes	Physiological and biochemical tests	Serum total protein, albumin, and transferrin
Urine 8-hydroxyguanosine (8-oxo-Gsn)	Yes	Yes		Yes	Physiological and biochemical tests	
Serum inflammatory factors	Yes	Yes		Yes	Physiological and biochemical tests	
Changes in serum differential metabolites	Yes	Yes			Utilize nontargeted metabolomics to assess serum metabolite changes pre- and post-intervention, elucidating the intervention’s mechanism	
Microbiota						
Changes in gut microbiota	Yes	Yes			Using the 16S sequencing method to detect changes in gut microbiota and differential bacterial genera among groups	
Comprehensive geriatric assessment						
Comprehensive geriatric assessment	Yes	Yes	Yes	Yes	Comprehensive Geriatric Assessment Questionnaire (CGAQ)	Physical assessment, functional assessment, medication review, social functioning assessment, environmental assessment, etc.
Intrinsic capacity						
Intrinsic capacity	Yes	Yes	Yes	Yes	Scale questionnaire	5-dimensional structure
State of health						
EuroQol Five Dimensions Questionnaire	Yes	Yes	Yes	Yes	EuroQol Five Dimensions Questionnaire	Mobility, self-care, usual activities, pain/discomfort, and anxiety/depression
Sleep conditions						
Pittsburgh Sleep Quality Index	Yes	Yes	Yes	Yes	Pittsburgh Sleep Quality Index	Rate the quality of sleep in the last 1 month
Participants’ satisfaction						
Participants’ satisfaction	Yes	Yes			Scale questionnaire	
Cognition function						
Cognition function	Yes	Yes	Yes	Yes	Mini-Mental State Examination (MMSE)	
Adverse event						
Adverse event	Yes	Yes	Yes	Yes		Serious, unexpected, and supervised adverse events

## Data Availability

The datasets to be used and/or analyzed are available from the corresponding author upon reasonable request.

## Supplementary Materials

The following supporting information can be downloaded at: https://www.mdpi.com/article/10.3390/nu17203213/s1, Supplemental Table S1: Anti-inflammatory diet recipe.

## Abbreviations

MINUTE: MultIfaceted NUtrition inTervention for frail Elderly (MINUTE) trial; 8-oxo-Gsn: 8-hydroxyguanosine; SPPB: Short Physical Performance Battery; Reps: Repetitions; CGAQ: Comprehensive Geriatric Assessment Questionnaire; MMSE: Mini-Mental State Examination; CRP: C-reactive protein; IL-6: interleukin-6; TNF-α: tumor necrosis factor-α; IGF-1: insulin-like growth factor-1; CXCL10: C-X-C-motif chemokine 10; CX3CL1: chemokine C-X3-C-based ligand 1; ICOPE: Integrated Care for Older People; IRB: institutional review board; SAEs: serious adverse events; ITT: Intention-to-Treat; PP: Per-Protocol; SD: standard deviation; IQR: interquartile range; CIs: confidence intervals; ORs: odds ratios; PAB: Patient Advisory Board.
