# Supplementary material for: Multifaceted Nutrition Intervention for Frail Elderly in the Community: Protocol of a Randomized Controlled Trial (The MINUTE Study)
Source: Nutrients. 2025 Oct 13;17(20):3213. doi: 10.3390/nu17203213 (PMC12566620; doi:10.3390/nu17203213)
Supplement: Supplementary file 1 [file nutrients-17-03213-s001.zip › nutrients-3906858-supplementary.pdf]

Supplemental Table S1. Anti-inflammatory diet menu

Anti-inflammatory Diet Menu						
**Date**	**Breakfast (7-9 AM)**	**Snack (9-10 AM)**	**Lunch (11-1 PM)**	**Snack (3-4 PM)**	**Dinner (5-7 PM)**	**Bedtime**
**Monday**	Whole wheat bread 100g<br>Nut butter<br>Unsweetened yogurt 100ml<br>Boiled egg 1	Nuts 25g	Quinoa brown rice<br>Lettuce beef<br>Kale scrambled eggs	Raspberries 200g	Black rice 1 bowl<br>Pan-fried salmon 120g<br>Stir-fried spinach 200g	22:00-22:30
**Tuesday**	Corn and millet porridge 100g<br>Skim milk 100ml<br>Kale scrambled eggs	Nuts 25g	Mixed rice<br>Celery stir-fried with beef<br>Stir-fried Shanghai greens	Cherry tomatoes 200g	Mixed rice 1 bowl<br>Green pepper stir-fried with shrimp 150g<br>Stir-fried Shanghai greens 200g	22:00-22:30
**Wednesday**	Boiled corn half<br>Unsweetened yogurt 100ml<br>Egg 1	Nuts 25g	Black rice and oat mixed grain rice<br>Green pepper stir-fried with chicken<br>Stir-fried flowering cabbage	Anti-inflammatory vitality drink: Cherry tomatoes | Banana | Avocado | Kale | Cucumber | Collagen peptide | Unsweetened coconut juice | Blender for 2 minutes	Boiled corn half<br>Wood ear stir-fried with chicken 150g<br>Stir-fried bok choy 200g	22:00-22:30
**Thursday**	Steamed sweet potato half<br>Skim milk	Nuts 25g	Red bean rice<br>Tomato stewed beef<br>Herb celery	Blueberries 200g	Oatmeal rice 1 bowl<br>Pan-fried salmon	22:00-22:30

	100ml<br>Chickpeas 20g<br>Egg 1		leaves<br>scrambled eggs		120g<br>Stir-fried Chinese cabbage 200g	
**Friday**	Red bean and oatmeal porridge 100g<br>Unsweetened yogurt 100ml<br>Herb celery leaves scrambled eggs	Nuts 25g	Oatmeal rice<br>Steamed sea bass<br>Stir-fried spinach	Anti-inflammatory vitality drink: Cherry tomatoes | Banana | Avocado | Kale | Cucumber | Collagen peptide | Unsweetened coconut juice | Blender for 2 minutes	Red bean and rice 1 bowl<br>Green pepper stir-fried with beef 150g<br>Stir-fried flowering cabbage 200g	22:00-22:30
**Saturday**	Berry oatmeal porridge<br>Buckwheat berry pancakes<br>Turmeric scrambled eggs	Nuts 25g	Steamed potato<br>Boiled shrimp<br>Stir-fried oil-soaked greens	Kiwi 200g	Purple sweet potato<br>Green pepper stir-fried with beef 150g<br>Stir-fried broccoli 200g	22:00-22:30
**Sunday**	Egg and vegetable buckwheat noodles<br>Low-fat milk	Nuts 25g	Barley brown rice<br>Mushroom stir-fried with chicken<br>Stir-fried lettuce	Grapes 200g	Boiled corn half<br>Pan-fried salmon 120g<br>Stir-fried hollow stem vegetables 200g	22:00-22:30
**Notes**	※<br>Quinoa brown rice: Quinoa 20g, brown rice 30g, steamed and served<br>Lettuce beef: Lettuce 80g, beef 60g, olive oil 10g<br>Stir-fried spinach: Spinach 150g blanched, add olive oil 10g, stir-fry over high heat for 1 minute					

	※<br>Mixed rice: Millet 15g, rice 30g<br>Celery stir-fried with beef: Beef 60g, celery 80g, olive oil 10g<br>Stir-fried Shanghai greens: Shanghai greens 100g, olive oil 10g<br>※<br>Boiled corn: Corn cob half<br>Green pepper stir-fried with chicken: Green pepper 70g, skinless chicken 80g (or chicken breast 70g), olive oil 10g<br>Stir-fried flowering cabbage: Flowering cabbage 100g, olive oil 10g<br>※<br>Red bean rice: Brown rice 30g, red bean 10g<br>Tomato stewed beef: Tomato 90g, beef 60g, olive oil 10g<br>Stir-fried Chinese cabbage: Broccoli 100g, cauliflower 100g, oil and vinegar dressing<br>※<br>Oatmeal rice: Oatmeal 20g, rice 20g<br>Steamed sea bass: Sea bass 80g, olive oil 10g<br>Stir-fried spinach: Spinach 100g blanched, add olive oil 10g, stir-fry over high heat for 1 minute<br>※<br>Steamed potato: Potato 100g<br>Boiled shrimp: Shrimp (prawn, green prawn, etc.) 80g<br>Stir-fried oil-soaked greens: Oil-soaked greens 100g, olive oil 10g<br>※<br>Barley brown rice: Barley 20g, brown rice 30g<br>Mushroom stir-fried with chicken: Mushroom 60g, skinless chicken 80g (or chicken breast 70g), olive oil 10g<br>Stir-fried lettuce: Lettuce 100g, olive oil 10g
